# Investigation of boron adsorption by graphene oxide: equilibrium, kinetic, and thermodynamic studies

**DOI:** 10.55730/1300-0527.3568

**Published:** 2023-05-27

**Authors:** Mehmet Fatih ÖNEN, Nalan ERDÖL AYDIN, Osman EKSİK, Pelin DEMİRÇİVİ, Gülhayat Nasun SAYGILI

**Affiliations:** 1 Department of Chemical Engineering, Faculty of Chemical and Metallurgical Engineering, İstanbul Technical University, İstanbul, Turkey; 2 Institute of Nanotechnology, Gebze Technical University, Gebze, Turkey; 3 Yalova University, Engineering Faculty, Chemical Engineering Department, Yalova, Turkey

**Keywords:** Graphene oxide, boron, adsorption, wastewater

## Abstract

Graphene oxide, which has a great application in industry, is one of the promising carbonous materials. Modified Hummers method was applied to synthesize graphene oxide. Characterization techniques showed that pure graphene oxide was successfully obtained. Its adsorptive properties were investigated by boron adsorption. The results were demonstrated that boron adsorption on graphene oxide was a pH-dependent process and maximum adsorption was achieved at pH 6 (0.98 mg g^−1^). Langmuir adsorption capacity was calculated as 3.92 mg g^−1^ with *R^2^* = 0.99. The kinetic data brought to light that pseudosecond-order kinetic model was well described the experimental data (*R^2^* = 0.99), and according to thermodynamic parameters, boron adsorption has spontaneous and endothermic nature (ΔH°*=*15.47 kJ mol^−1^).

## 1. Introduction

Boron mineral is an important micronutrient for humans, animals, and plants. However, its concentrations were restricted due to harmful effects. Boron concentration is restricted as 0.5 mg L^−1^ in irrigation water and 2.4 mg L^−1^ in drinking water by World Health Organization (WHO) [1]. Due to application of boron in various industries, such as glass, ceramics, detergent, semiconductors, and cosmetics, boron concentration in wastewaters is a serious problem.

Removal of boron from wastewater has been studied using coagulation and coprecipitation process [2, 3], adsorption process [4–6], ion exchange process [7–9], solvent extraction process [10], and membrane processes [11–13].

Adsorption is the most useful technique among boron removal techniques due to being applicable at low concentrations with high adsorption capacities. Boron adsorption was studied using various adsorbents. Chen et al. examined boron adsorption using magnetic magnetite (Fe_3_O_4_) nanoparticles with the maximum adsorption capacity as 4.57 mmol g^−1^ at pH 7 [5]. Jung et al. investigated boron adsorption using polystyrene-based resin utilizing its chelating properties with boron and obtained two times greater adsorption capacity [4]. Kluczka et al. studied commercial and modified activated carbon for boron removal from wastewater. Polyhydric chelates were used for modification of commercial activated carbon and maximum adsorption capacity was found as 1.50 mg g^−1^ [14]. Boron removal was also studied by clay minerals such as vermiculite and perlite in our previous studies. Vermiculite and perlite clays were modified with hexadecyltrimethylammonium bromide (HDTMA) and gallic acid to increase the adsorption capacities of the clay minerals due to electrostatic interactions and chelating properties of boron [15, 16].

Graphene is a new carbon-based material with high surface area and excellent mechanical properties [17]. Graphene oxide is synthesized by oxidation of graphene. Graphene oxide includes various oxygen functional groups, such as hydroxyl, epoxy, and carboxyl groups [18]. These oxygen groups in graphene oxide structure promote the binding ability of the adsorbate molecule [19]; therefore, graphene oxide is an effective adsorbent to remove boron from wastewater. Graphene oxide was generally used for the removal of metal ions, such as arsenic and mercury [20]. Zhao et al. studied cadmium and cobalt adsorption [19]. Wu et al. examined copper removal by graphene oxide [21].

In this study, raw graphene oxide was used for boron adsorption to establish its usability and adsorption capacity for removal of boron. Modified Hummers method was used to synthesize graphene oxide in our research laboratory and characterization was performed with various methods. Synthesized graphene oxide was used as an adsorbent for boron adsorption to investigate the effects of adsorbent amount, solution pH, initial boron concentration, contact time, and temperature. Obtained data was used to determine the isotherm, kinetic, and thermodynamic parameters.

## 2. Materials and methods

### 2.1. Chemicals and reagents

Graphite powder was purchased from Grafen Chemical Industries (Grafen Co.). Potassium persulfate (99.99%), phosphorus pentoxide (98.0%), potassium permanganate (99.0%), hydrogen peroxide (30%), ascorbic acid (99.0%), ammonium acetate (99.99%), acetic acid (100%), boric acid (99.5%), and ethylenediaminetetraacetic acid disodium salt (99.0%) were obtained from Merck, Germany. Azomethine-H reagent (97.0%) was purchased from Acros Organics, Belgium. Thyoglycolic acid (80.0%) was obtained from Sigma-Aldrich, USA. All chemicals used in the experiments were in analytical grade.

### 2.2. Synthesis of graphene oxide

Modified Hummers method was used to synthesize graphene oxide from graphite [22]. First, 240 mL of 98% H_2_SO_4_ was added slowly onto a certain amount of preoxidated graphite. To solve the mixture, 30 g of KMnO_4_ was put into the solution while keeping the temperature constant at 10 °C. The temperature was then raised to 35 °C and the mixture was mixed for 4 h. Next, 500 mL of distilled water was added drop by drop into the solution and agitated for 1 h. Subsequently, 40 mL of 35% H_2_O_2_ was added into the remaining solution. The solution was aged for 1 day and filtered. The mixture was washed with 10% HCl solution and acetone until neutral pH was reached. Synthesized graphite oxide was dried in an oven at 60 °C. Finally, graphite oxide was ultrasonicated for 2 h, and after centrifugation, graphene oxide was synthesized and dried in an oven at 60 °C.

### 2.3. Characterization of materials

The materials were thoroughly characterized through the utilization of advanced analytical techniques. Specifically, X-ray diffraction analysis was conducted utilizing the state-of-the-art PANanlytical X’Pert PRO diffractometer, providing high precision and accuracy in crystal structure determination. Fourier transform infrared spectrometry was employed using the Perkin Elmer Spectrum One instrument to identify and analyze functional groups present in the material. Additionally, scanning electron microscopy using the cutting-edge FEI QUANTA FEG 250 was employed to obtain high-resolution images and information regarding the surface morphology of the material. This comprehensive characterization approach allowed for a thorough understanding of the properties and characteristics of the materials studied.

### 2.4. Boron adsorption experiments

Boron removal was studied in batch system and various parameters such as adsorbent amount, solution pH, initial solution concentration, contact time and temperature were examined. To investigate the effect of adsorbent dosage, various amounts of graphene oxide (0.05–0.4 g) was mixed continuously with 50 mL boron solution (4 mg L^−1^) on a mechanical shaker. Determined amount of graphene oxide was used to examine the pH effect on boron adsorption by changing solution pH between 2 and 12. The pH of the solutions was adjusted with 0.1 M HNO_3_ and 0.1 M NaOH solutions. The effect of initial boron concentration was studied by shaking a series of 50 mL boron solution whose concentrations were changed between 2 and 36 mg L^−1^. Kinetic experiments were conducted at different time intervals (1–36 h) by shaking 50 mL of 4 mg L^−1^ boron solution with 0.2 g graphene oxide. Thermodynamic experiments were studied at 298, 308, and 318 K to investigate the thermodynamic parameters.

### 2.5. Determination of boron concentration

Boron concentration remaining in the solution was determined by Azomethine-H method and measured by UV-Vis spectrophotometer (Jenway 6305, UK). The adsorption capacity of graphene oxide was calculated by the following equation;


(1) $$q_{e}=\frac{\left(C_{o}-C_{e}\right)}{m}*V,$$

where *q_e_* represents the adsorption capacity at equilibrium (mg g^−1^), *C_o_* shows the initial boron concentration, and *C_e_* is the equilibrium concentration of boron solution (mg L^−1^), *m* is the amount of graphene oxide (g), and *V* is the solution volume (L).

## 3. Results and discussion

### 3.1. Characteristic properties of graphene oxide

**Figure 1:**
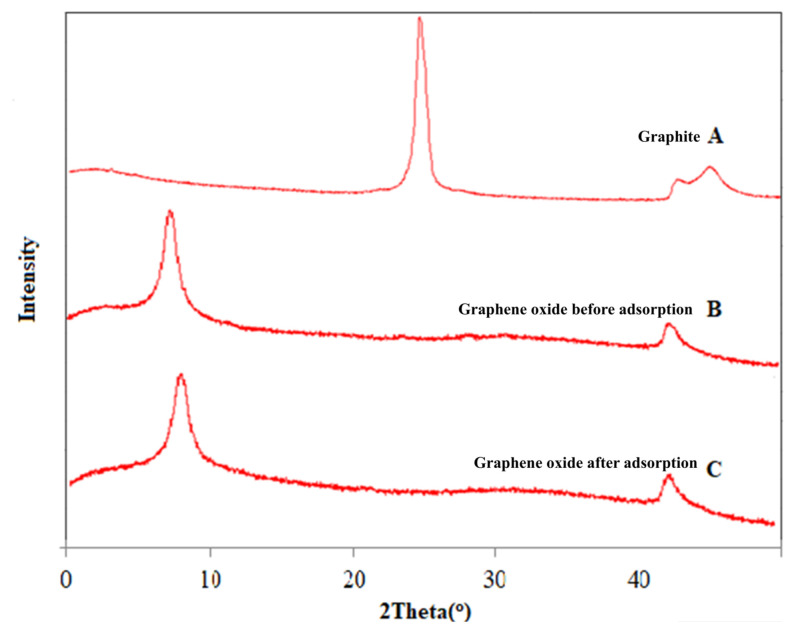
X-ray diffraction (XRD) patterns of graphite, graphene oxide before adsorption, and graphene oxide after adsorption.

X-ray diffraction patterns of graphite, graphene oxide before adsorption, and graphene oxide after adsorption are shown in Figure 1. Graphite showed a strong peak at 2θ = 27.2° that is characteristic peak of graphite [23]. The diffraction peak at 2θ = 11.6° assigned the raw graphene oxide (before adsorption) peak and found appropriate as in the literature, which indicates forming of graphene oxide layers from graphite. *d*-spacing of graphite and graphene oxide was 0.328 nm and 0.759 nm, respectively. Increasing *d*-spacing between the layers can be attributed to the bounding of oxygen groups into the structure while synthesizing the graphene oxide from graphite. XRD pattern of graphene oxide after boron adsorption was similar to raw graphene oxide, which might be attributed to the low boron concentration used in the experiments.

**Figure 2:**
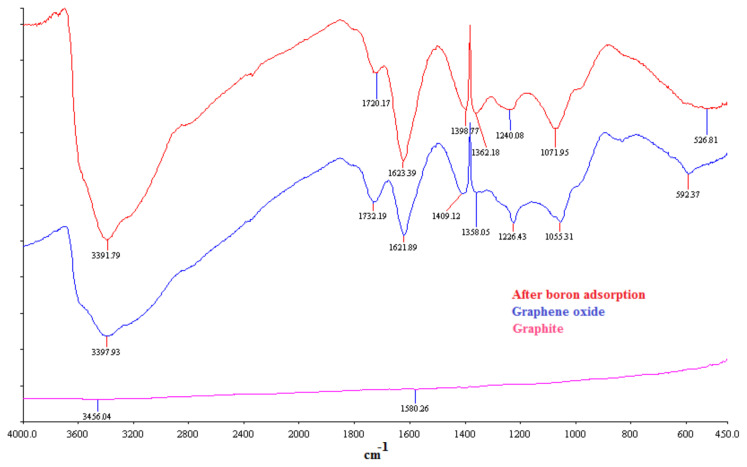
FTIR analysis of graphite, graphene oxide, and graphene oxide after boron adsorption.

FTIR spectra of graphite and graphene oxide are given in Figure 2. FTIR spectra of graphite displayed a straight line due to polyaromatic layers in graphite structure. FTIR spectra of graphene oxide exhibited characteristic peaks. Graphene oxide spectra showed a wide peak at 3340 cm^−1^, indicating vibration band of OH group of water bounded on the structure [19]. The absorption peaks at 1732 cm^−1^ and 1620 cm^−1^ could be attributed to vibration band of C=O group of carboxylic acid and C=C groups in graphene oxide structure [20]. The peak between 1055 and 1230 cm^−1^ assigned to vibration band of C-O belongs to epoxy and alkoxy groups. Stretching vibration of S-O group that appeared at 831 cm^−1^ belongs to sulfur in graphene oxide structure. FTIR spectra after boron adsorption did not show any significant change with the raw graphene oxide.

**Figure 3:**
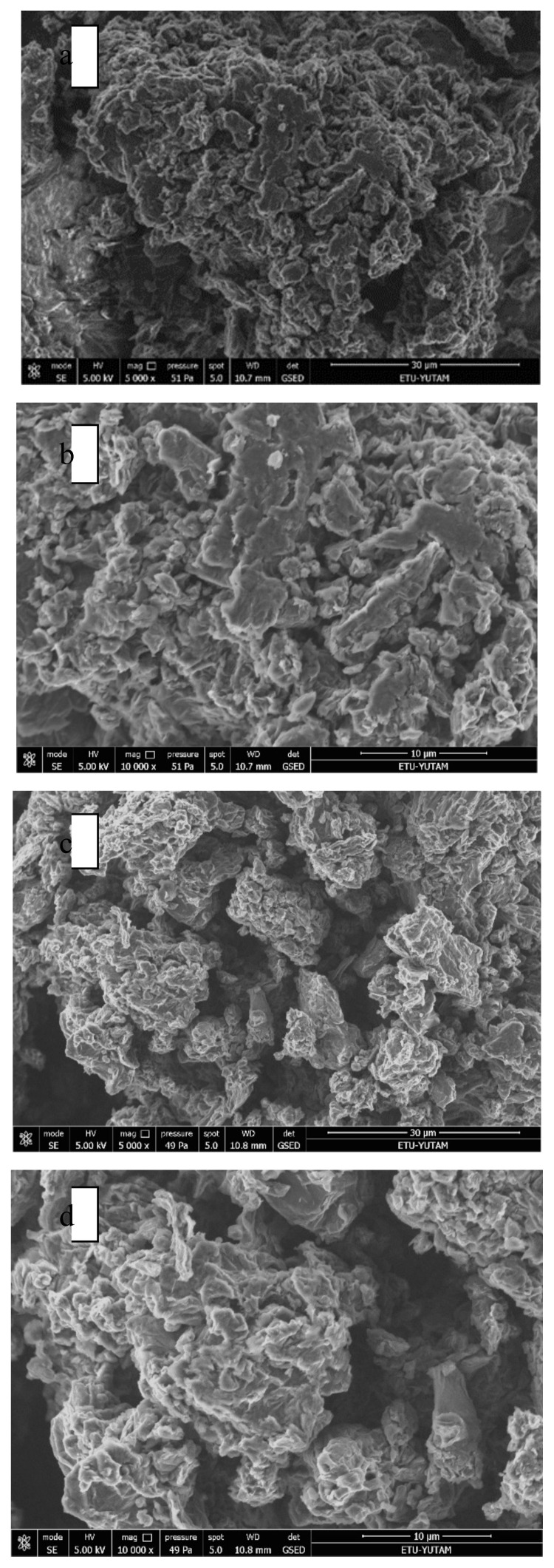
SEM-EDX analysis of graphene oxide before adsorption (a) 5000×, b) 10,000×) and after boron adsorption (c) 5000×, d) 10,000×).

SEM images and EDX analysis of all samples are given in Figure 3. As shown in the graph, it was observed that the surface of graphite was rough and edges of the layers were sharp. After the oxidation process, graphene oxide exhibited a porous structure and some folds, which indicated graphene oxide existence. Figure 3 shows the graphene oxide surface morphology after adsorption, which did not display any difference with raw graphene oxide due to low concentration of boron. Meanwhile, according to EDX results, C and O contents in graphite and graphite oxide structures were in a good agreement.

### 3.2. Graphene oxide amount

**Figure 4:**
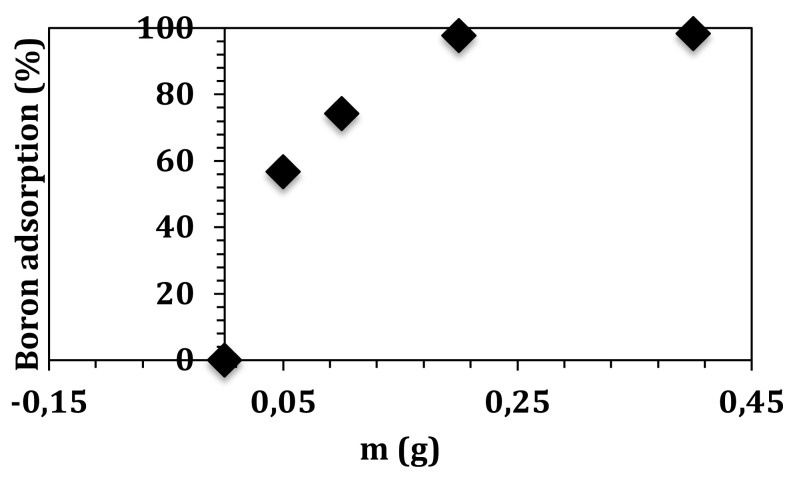
Effect of graphene oxide amount on boron adsorption.

The influence of adsorption dosage was investigated by changing graphene oxide amount between 0.05 and 0.4 g using 4 mg L^−1^ of initial boron concentration. Figure 4 shows the change of boron removal with graphene oxide amount. Boron adsorption rapidly increased from 56% to 98% for 0.05 g and 0.2 g graphene oxide, respectively. A significant change was not observed using 0.4 g of graphene oxide (98.3%), which indicates that the active adsorption sites was fully occupied by boron molecules. Therefore, 0.2 g of graphene oxide was used for the further adsorption experiments. Meanwhile, a decline was achieved for the adsorption capacity, which can be attributed to the gathering of the graphene oxide molecules by intermolecular interactions.

### 3.3. Solution pH

**Figure 5:**
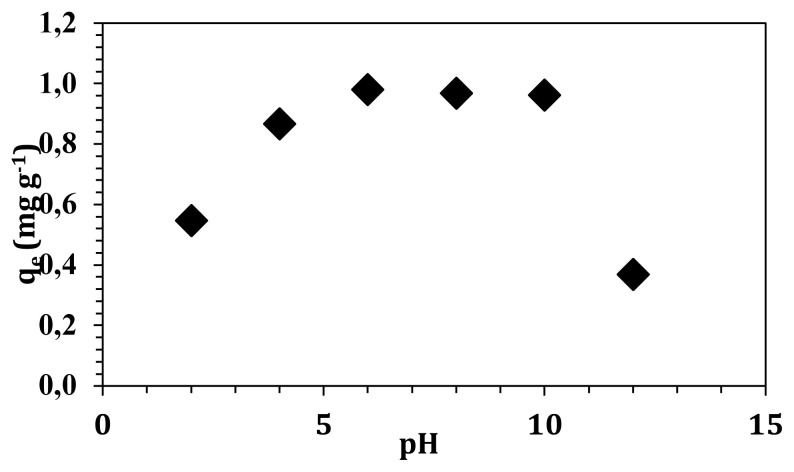
pH-dependent adsorption capacity of graphene oxide.

Solution pH is an important factor that describes the surface properties of the adsorbent and also molecular structure of the adsorbate. Therefore, solution pH was adjusted from pH 2 to 12, and the adsorption capacities are given in Figure 5.

As seen from Figure 5, maximum adsorption capacity was achieved at pH 6 (0.98 mg g^−1^), a slight decrease was observed until pH 10 (0.96 mg g^−1^), and a sharp decrease was seen at pH 12 (0.37 mg g^−1^). Boric acid forms borate anion in water with p*K_a_* value 9.2. According to the solubility reaction below pH 8, boric acid (H_3_BO_3_) is the dominant species in water, while borate anion (B(OH)_4_^−^) is the dominant species above pH 10. Between pH 8 and 10, occurrence of H_3_BO_3_ species diminishes slowly and B(OH)_4_^−^ species rises in water medium. Besides, hydroxyl and carboxylic acid groups on graphene oxide structure are protonated in acidic medium and the surface charge becomes positive. As the pH increases, the surface loses its protons and the surface becomes negatively charged. According to ionization of boric acid and protonation of graphene oxide surface, hydrogen bonding between oxygen and hydrogen molecules is the significant reason for the adsorption of boric acid in acidic medium. Boric acid slowly decreases because the borate anion begins to form in the solution as the pH increases, which causes a higher adsorption of boron. After pH 10, a significant decrease was observed due to electrostatic repulsive forces between the B(OH)_4_^−^ and negatively charged surface of graphene oxide. Besides, the competition between hydroxyl molecules (OH^−^) and B(OH)_4_^−^ anions had a negative effect on boron adsorption; therefore, a sharp decrease was observed after pH 10.

### 3.4. Initial boron concentration and adsorption isotherms

**Figure 6:**
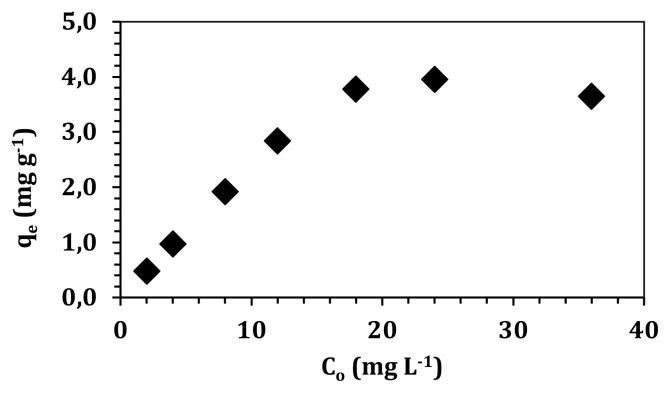
Initial concentration effect on boron adsorption.

One of the parameters that affect adsorption capacities of graphene oxide is initial solution concentration. Boron solution concentrations were changed between 2 and 36 mg L^−1^, and experiments were conducted using 0.2 g of graphene oxide at room temperature during 24 h. As shown in Figure 6, at low boron concentrations, 98% adsorption was achieved (4 mg L^−1^) and it decreased to 40% for 36 mg L^−1^ boron concentration. Certain amount of active adsorption sites was occupied with boron molecules, and after a point, no further adsorption occurred on these sites. Therefore, it was easy to adsorb boron at low concentrations due to the number of free adsorption sites. However, increasing boron concentration resulted in a decrease in adsorption, because all adsorption sites were covered by a certain amount of boron molecules and the rest of the boron molecules remained in the solution.

**Figure 7:**
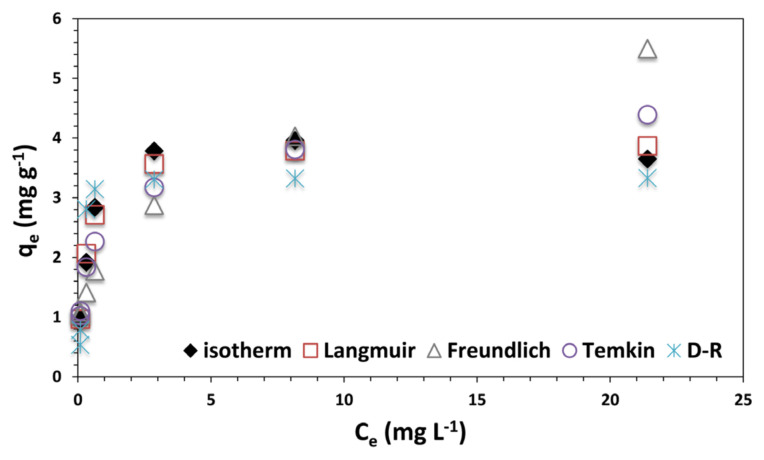
Isotherm models of graphene oxide for boron adsorption.

According to initial solution parameters, adsorption isotherm and isotherm models were analyzed. Figure 7 shows the isotherm of boron adsorption on graphene oxide surface. According to Giles et al. (1960), isotherm curves are classified into four main groups and each of them has subgroups [24]. Isotherm graph of boron adsorption defines L type isotherm model and belongs to *L2* type subgroup. *L* type isotherms define Langmuir isotherms, which implies monolayer adsorption on graphene oxide.

Langmuir, Freundlich, Temkin, and Dubinin-Redushkevich isotherm models were used to analyze the isotherm data [16]. Langmuir isotherm model describes the coenergy adsorption sites on the adsorbent surface, and as a result, monolayer adsorption occurs on the same energy sites [25]. Freundlich isotherm explains heterogeneity of the surface that has different energy sites. Temkin isotherm model defines the reduction of adsorption heat, which is linear. Dubinin-Radushkevich isotherm model depends on the porosity of the adsorbent and takes into account the mean free energy of the adsorption.

To identify the conformity of isotherm models and experimental data, correlation coefficient and chi-square values were compared. To calculate the chi-square values, the equation below was used:


(8) $$X^{2}=\sum \left[\frac{{\left(q_{cal}-q_{exp}\right)}^{2}}{q_{cal}}\right]$$

The parameters calculated from the isotherm data are given in Table 1. Small values of *X^2^* indicate that the isotherm model is in a good agreement with experimental data. According to correlation coefficients (*R^2^*) and chi-square (*X^2^*) values, Langmuir isotherm model (*R^2^* = 0.99, *X^2^* = 0.049) was best-fitted model of adsorption process, which indicates the monolayer adsorption of boron on graphene oxide. Langmuir adsorption capacity was found as 3.92 mg g^−1^. As shown in Figure 7, calculated *q_e_* values were in a good correlation with those calculated from Langmuir model among all isotherm models. Ismonto et al. (2014) studied synthesis of activated carbon and carbon nanotubes for boron removal from wastewater. Carbon nanotubes were modified with polyvinyl alcohol (PVA) and adsorption capacity was found as 1.19 mg g^−1^, while it was 1.28 mg g^−1^ for unmodified carbon nanotubes. Besides, Langmuir isotherm model was the best-fitted model for boron adsorption [26]. Zohdi et al. synthesized multiwalled carbon nanotubes, which was exhibited 1.97 mg g^−1^ adsorption capacity for boron removal [27]. Jaouadi et al. synthesized activated carbon from pinewood sawdust from timber industry, and boron adsorption occurred according to monolayer adsorption with 1.42 mg g^−1^ adsorption capacity [28].

Dimensionless constant separation factor (*R_L_*) can be calculated using Langmuir constant *b* (L mg^−1^). *R_L_* defines if the adsorption process is favorable or unfavorable. *R_L_* is calculated as:


(9) $$R_{L}=\frac{1}{1+bC_{0}}.$$

**Figure 8:**
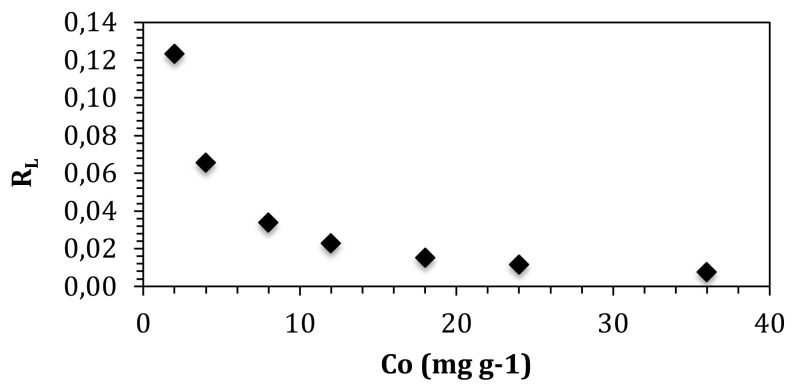
Separation factor according to initial boron concentration.

Calculated *R_L_* dimensionless separation factor is defined as *R_L_* > 1 unfavorable *R_L_* = 1 linear, 0 < *R_L_* < 1 favorable, and *R_L_* = 0 irreversible. Correlation between initial solution concentration and dimensionless separation factor is depicted in Figure 8. *R_L_* values of boron adsorption process changed between 0 and 1. Therefore, it is clear that adsorption of boron on graphene oxide was a favorable adsorption. The results are in a good agreement with the literature search [29, 30].

### 3.5. Contact time and adsorption kinetics

**Figure 9:**
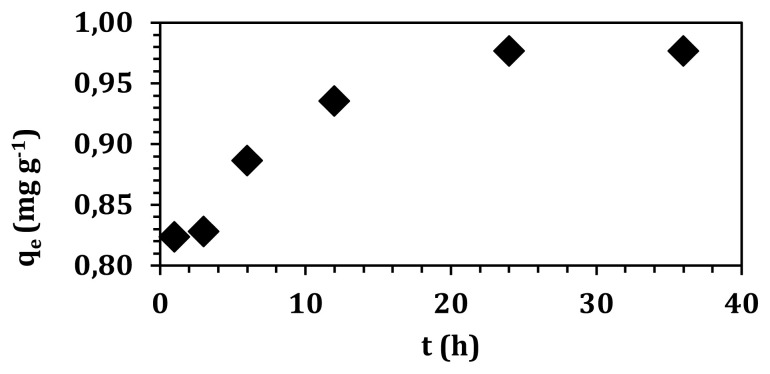
Kinetics of boron adsorption onto graphene oxide.

Contact time experiments were examined between 1 and 36 hours using 4 mg L^−1^ of initial boron concentration. Until 3 h, no remarkable change was observed on boron adsorption (Figure 9). However, after that point, a significant increase was achieved. A plateau was reached between 24 h and 36 h, which attributed the adsorption equilibrium.

The kinetic modeling of boron adsorption was analyzed using various kinetic models [31]. Kinetic model parameters were calculated and given in Table 2. Pseudosecond-order kinetic model was the best-fitted model among four models when the correlation coefficients were compared (*R^2^*=0.99). Pseudosecond-order kinetic model was derived based on three assumptions: (i) the adsorption process is irreversible; therefore, desorption can be neglected, (ii) there is no concentration gradient of the adsorbate remaining in the solution, (iii) adsorption process is controlled by a chemical reaction [32, 33]. Meanwhile, calculated adsorption capacity from pseudosecond-order kinetic model (0.992 mg g^−1^) demonstrated a good correlation with the experimental value (0.986 mg g^−1^). According to the literature comparison, pseudosecond-order kinetic model was best fitted for boron adsorption [34,35].

### 3.6. Adsorption thermodynamics

To understand the effect of temperature on boron adsorption, batch adsorption experiments were examined at 298, 308, and 318 K. It was observed that increasing temperature has a positive effect on boron adsorption on graphene oxide. Meanwhile, thermodynamic parameters were also calculated to better understand the adsorption behavior.

Change in standard enthalpy (Δ*H*°) was calculated according to Van’t Hoff equation [36];


(14) $$ln\;\left(\frac{C_{e2}}{C_{e1}}\right)=\frac{\Delta H^{o}}{R}\left(\frac{1}{T_{2}}-\frac{1}{T_{1}}\right).$$

Change in standard Gibbs free energy (ΔG°) was given as [34];


(15) $$\Delta G^{o}=-RTlnK,$$

(16) $$K=\frac{C_{ads}}{C_{e}}$$

Change in standard entropy (ΔS°) was calculated as [34];


(17) $$\Delta G^{o}=\Delta H^{o}-T\Delta S^{o}$$

Calculated thermodynamic parameters are given in Table 3. Positive value of ΔH° pointed out that the adsorption process has endothermic nature. Equilibrium constant values (*K*) showed a good correlation with ΔH°. Increasing temperature resulted in an increment in *K* values. ΔG° values were found negative suggesting that the boron adsorption process spontaneously occurred at all temperatures. Positive values of ΔS° indicated that randomness at solid/liquid interface and degree of freedom of the adsorbed boron were increased.

## 4. Conclusion

Boron adsorption was studied using graphene oxide, which was synthesized by modified Hummers method and the structure of graphene oxide was lightened. According to XRD analysis, graphene oxide was obtained successfully from graphite that gave the characteristic peak at 2θ = 11.6°. Meanwhile, FTIR analysis proved the synthesized graphene oxide C=O, C-O, and C=C bands. Boron adsorption strongly depended on the solution pH and the maximum adsorption capacity was found at pH 6 with q_e_= 0.98 mg g^−1^. Experimental data was well correlated with Langmuir model (q_max_= 3.92 mg g^−1^ and *R^2^* = 0.99), which indicates monolayer adsorption of boron on graphene oxide surface. Electrostatic attractions and chelating ability of boron were responsible forces for boron adsorption. Kinetic data followed pseudo-second-order kinetic model (*R^2^* = 0.99), and according to thermodynamic parameters, boron adsorption had spontaneous and endothermic nature. All the results given above indicated that graphene oxide could be used as a promising adsorbent for the adsorption of boron from wastewater.

## Figures and Tables

**Table 1:** Isotherm parameters for boron adsorption by graphene oxide.

Langmuir isotherm					Freundlich isotherm			
Q_m_ (mg g^−1^)	b	R^2^	*X* *^2^*	K_F_ (mg g^−1^)	n	R^2^	*X* *^2^*	
3.92	3.55	0.99	0.049	2.05	3.11	0.75	2.07	
**Temkin isotherm**				**Dubinin-Reduskevich isotherm**				
**K** **_t_** ** (L g** **^−1^** **)**	**b**	**R** **^2^**	***X*** *^2^*	**q** **_m_** ** (mg g** **^−1^** **)**	**B (mmol** **^2^** ** kJ** **^−2^** **)**	**E (kJ mol** **^−1^** **)**	**R** **^2^**	***X*** *^2^*
69.99	0.60	0.87	0.70	3.37	0.017	0.75	0.93	0.58

**Table 2:** Kinetic paratemeters for boron adsorption by graphene oxide.

Pseudofirst-order model			Pseudosecond-order model		
k_1_ (L min^−1^)	q_e_ (mg g^−1^)	R^2^	k_2_ (g mg^−1^ min^−1^)	q_e_ (mg g^−1^)	R^2^
0.0021	0.19	0.97	0.032	0.99	0.99
**Intraparticle diffusion model**			**Elovich model**		
**k** **_int_** ** (mg g** **^−1^** ** min** **^−1/2^** **)**	**c**	**R** **^2^**	α (g mg**^−1^ min^−1^)**	β (g mg**^−1^)**	**R** **^2^**
0.0055	0.77	0.96	1.75	0.052	0.93

**Table 3:** Thermodynamic parameters for boron adsorption by graphene oxide.

T (K)	ΔH (kJ mol^−1^)	ΔG (kJ mol^−1^)	ΔS (J mol^−1^ K^−1^)
298		−9.27	
308	15.47	−10.10	83.02
318		−10.93	
